# Winter Nursing Engagements

**Published:** 1888-03-17

**Authors:** 


### WINTER NURSING ENGAGEMENTS.

A nurse willing to go out to India to nurse under Government should apply to the Under-Secretary of State for India, Whitehall, London.

